# P33 The successful use of Belimumab in recurrent small bowel obstruction secondary to Lupus Enteritis

**DOI:** 10.1093/rap/rkac067.033

**Published:** 2022-09-28

**Authors:** Amanda Mootoo, Mark Gibson, Begoña Lopez

**Affiliations:** Guy's and St Thomas' Hospital NHS Foundation Trust, London, United Kingdom; Guy's and St Thomas' Hospital NHS Foundation Trust, London, United Kingdom; Guy's and St Thomas' Hospital NHS Foundation Trust, London, United Kingdom

## Abstract

**Introduction/Background:**

Lupus enteritis encompasses mesenteric vasculitis, enteric vasculitis, mesenteric arteritis and lupus peritonitis and is an uncommon, potentially life-threatening presentation of SLE. The prevalence in SLE patients ranges from 0.2% to 9.7% and up to 65% in SLE patients presenting with acute abdominal pain. This case brings awareness to the gastrointestinal manifestations of lupus with particular focus on the clinical and radiological features of SLE-related vasculitis of the gastrointestinal tract. It highlights the importance of recognising new ascites and hydronephrosis as a presenting feature of LE with leucopenia, low C3 and raised IgA as additional associated features.

**Description/Method:**

A 48-year-old female presented to hospital abdominal pain and vomiting. Abdominal CT imaging revealed small bowel obstruction with extensive mural oedema and thickening of the ileum, jejunum, ascending, transverse and descending colon. Ryle’s tube was inserted and left on free drainage. Imaging also showed ascites, bilateral hydronephrosis bilateral hydronephrosis, enlarged pelvic lymph nodes, pleural effusions and a small pericardial effusion.

Past medical history included seronegative, anti-CCP positive rheumatoid arthritis for which she was on taking baricitinib 4mg daily and prednisolone 5mg daily. Otherwise she had a history of HbSC disease with previous delayed transfusion reaction, urticarial vasculitis, fully treated tuberculosis.

This was the patient’s 3rd presentation with recurrent abdominal pain, vomiting and small bowel obstruction in the preceding last 6 months. 6 weeks prior to the current presentation she underwent a laparotomy, revealing a chronic dilated ∼40cm segment of small bowel and ∼800ml ascites. Histology revealed normal mucosa with no evidence to support inflammatory bowel disease, tuberculosis or infective colitis, but was significant for sub-mucosal fat necrosis with foamy histiocytes present.

On this occasion, the clinical picture deteriorated with the development of hospital acquired pneumonia, acute kidney injury (creatinine 170, urine protein:creatinine ratio of 615) and fluid overload (albumin 27) requiring admission to intensive care unit.

Immunology results revealed ANA 1/2560 speckled, anti-dsDNA 72 IU/ml, Ro-60 positive, RNP 70 positive, C3 0.51 (ref range (0.90-1.80 g/L), C4 0.18 (ref range 0.10 – 0.40 g/L), rheumatoid factor <10, anti-CCP 154 (ref range (0-7IU/ml).

A diagnosis of systemic lupus erythematosus (SLE) with lupus enteritis and probable lupus nephritis was made. Pulsed methylprednisolone and belimumab therapy were commenced followed by the addition of mycophenolate mofetil resulting in sustained improvement in clinical, biochemical and radiological parameters and no further episodes of bowel obstruction.

**Discussion/Results:**

LE is an uncommon but potentially life-threatening complication of SLE. Symptoms for LE can vary from mild intermittent diffuse abdominal pain, nausea, vomiting, bloating, diarrhoea to acute abdominal pain, with urinary symptoms present in up to 20%. LE is most likely to occur in patients with high disease activity, determined by disease activity measures such as the BILAG or SLEDAI. Abdominal computer tomography (CT) is the gold standard investigation with the typical features being diffuse or focal bowel wall thickening and oedema (‘target sign’), mesenteric vasodilation (‘comb sign’), dilatation of intestinal segments and ascites. Endoscopy may show ischaemia and ulcerative changes, but biopsies are often normal. Full thickness biopsies may be required. Urinary tract involvement and hydronephrosis have been described in association with LE.

There are currently no specific available guidelines or recommendations for the treatment of LE. Rapid improvement is seen following administration of high-dose glucocorticoids and additional immunosuppression using Cyclophosphamide, Rituximab Azathioprine or Mycophenolate mofetil can be used in certain cases. The relapse rate is common with predictors of recurrence reported as bowel wall thickness exceeding 9mm and those receiving glucocorticoids alone. Potentially life-threatening complications including gastro-intestinal perforation and intestinal necrosis if left untreated.

Based on the positive lupus serology, extensive bowel oedema, ascites, and bilateral hydronephrosis in addition to lymphadenopathy, pleural effusions and small pericardial effusion seen on CT imaging; LE was the top differential in this case. High dose intravenous steroids with the addition of Belimumab were promptly initiated in view of the rapid accumulation of ascites, progression of hydronephrosis and deterioration in renal function.

**Key learning points/Conclusion:**

This case highlights the importance of recognising new ascites and hydronephrosis as a presenting feature of LE with leucopenia, low C3 and raised IgA as additional associated features.

The timely diagnosis of LE is crucial to preventing potentially life-threatening complications including gastrointestinal perforation and intestinal necrosis if left untreated.

This case explores the importance of distinguishing LE from other differentials such as infectious colitis, TB enteritis, inflammatory bowel diseases.

The diagnosis of LE can be determined through imaging studies, specifically abdominal CT scans. This case demonstrates that the typical CT findings of bowel oedema, thickening, ascites and hydronephrosis was key is identifying the diagnosis of LE.

Early identification is crucial in LE to enable the timely administration of high-dose glucocorticoids, as LE is typically steroid-responsive with an overall good prognosis once treated.

Although there is no specific immunological test to confirm a diagnosis of LE, it is essential to recognise that this constellation of clinical features: ascites, hydronephrosis, leucopenia, low C3 and raised IgA, are independently associated with LE and should prompt a potential diagnosis of LE.

Whilst the decision for further immunosuppression following glucocorticoid may be made on an individualised basis, this case of LE demonstrates that prompt treatment with monthly intravenous Belimumab is a suitable immunosuppressive option.

Prompt immunosuppression provided rapid symptomatic benefit and reversal of bowel oedema and hydronephrosis on repeat imaging.

As of yet, this is the first reported case in the literature whereby Belimumab was successfully used to treat lupus enteritis. Future studies are however needed to explore this further.

